# Myelopathy from Intradural Extramedullary Metastasis as an Initial Presentation of Metastatic Melanoma

**DOI:** 10.7759/cureus.2668

**Published:** 2018-05-22

**Authors:** Alan A Stein, Gila R Weinstein, Colin Niezgoda, Sajeel Chowdhary, Frank Vrionis, John K Houten

**Affiliations:** 1 Boca Raton Regional Hospital, Marcus Neuroscience Institute, Boca Raton, USA; 2 Division of Plastic Surgery, Maimonides Medical Center; 3 Department of Neurosurgery, Marcus Neuroscience Institute, Boca Raton, USA; 4 Neuro-Oncology, Marcus Neuroscience Institute, Boca Raton, USA; 5 Department of Neurological Surgery, Marcus Neuroscience Institute, Boca Raton, USA; 6 Neurosurgery, Maimonides Medical Center

**Keywords:** intradural extramedullary tumor, malignant melanoma, myelopathy, spinal metastasis, spinal tumor

## Abstract

The incidence of metastatic melanoma (MM) has been steadily rising, and it is the third most common metastatic lesion to the central nervous system (CNS). Spinal intradural extramedullary (IDEM) MM is rare, and it is associated with coexisting or antecedent brain metastasis. Metastatic disease to the CNS is a complication of advanced disease, and it generally occurs months to years after initial diagnosis and treatment. We describe the first case of an initial presentation of MM, presenting as cervical myelopathy secondary to spinal cord compression from IDEM spinal metastasis. Further work-up revealed additional lesions in the temporal lobe and cauda equina region as well as a scalp lesion that was presumed to be the primary site. MM should be considered in the differential of myelopathy secondary to a spinal intradural mass, particularly in those with a history of or risk factors for melanoma.

## Introduction

The incidence of malignant melanoma (MM) has been steadily rising, and it is the third most common cause of central nervous system (CNS) metastases [1]. Metastasis to the CNS is a common, yet serious, complication of advanced disease in patients with melanoma and carries a poor prognosis. Several studies have reported median overall survival of only four to six months from the time of diagnosis of MM to the CNS [2-4]. Metastatic CNS disease is a late complication of advanced melanoma and generally presents months to years after initial diagnosis and treatment. Only about seven percent of patients with melanoma have brain metastases at the time of diagnosis [5]. Metastatic deposits of tumor in the spinal intradural extramedullary (IDEM) space are rare [6], and they are typically found in patients with antecedent or coexisting cerebral metastasis [7]. We describe the first case in which a presentation of cervical myelopathy secondary to spinal cord compression from intradural extramedullary spinal metastasis was the initial presentation of MM.

## Case presentation

A 63-year-old morbidly obese male presented with a three-month course of progressively worsening symptoms of neck pain, inability to walk, and numbness and weakness in the distal upper extremities. Neurological examination demonstrated 4/5 strength in the triceps and hand intrinsics, 2/5 in the iliopsoas, and nondermatomal distal upper extremity sensory loss with an upper thoracic pin level. Deep tendon reflexes were diminished, but Babinski and Hoffmann signs were bilaterally present. Later in the hospital stay, a subtle left-sided visual field deficit was appreciated.

**Figure 1:**
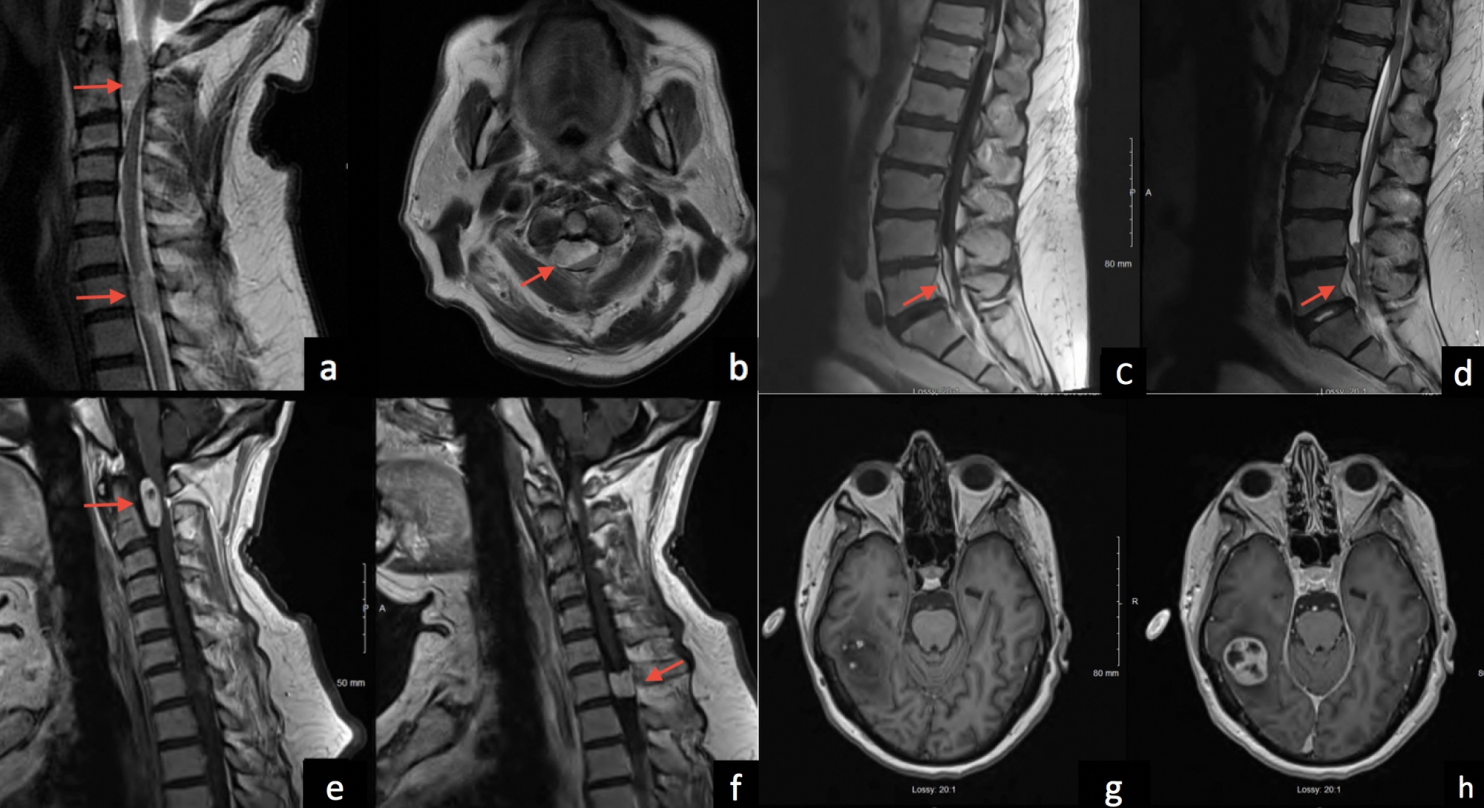
Magnetic resonance imaging of the cervical and lumbar spine and brain. T2-weighted magnetic resonance imaging (MRI) of the cervical spine in sagittal (a) and axial (b) planes, the latter at the C2 level, demonstrating intradural extramedullary lesions with compression of the spinal cord. The lesions are seen to brightly enhance on T1-weighted sagittal images with intravenous contrast (e-f). Sagittal T1 contrast-enhanced (c) and T2-weighted (d) MRI of the lumbar spine showing intradural extramedullary masses at the conus medullaris and L4-S1 levels. Axial MRI of the brain precontrast (g) and postcontrast (h) demonstrates an approximately 3 cm enhancing mass in the posterior right temporal region with perilesional edema.

Magnetic resonance (MR) imaging revealed an enhancing IDEM 1.5 cm × 1.0 cm mass at C2 causing severe cord compression. A similar lesion was noted at the C7 level (Figure 1). Subsequent MR of the remainder of the neuroaxis revealed a 3 cm right-sided posterior temporal lobe mass with small hemorrhagic foci and surrounding edema and matting of the roots of the cauda equina (Figure 1).

A systemic work-up revealed a scalp lesion over the right ear which was subsequently shown on biopsy to be a melanoma, but without any visceral metastasis.

**Figure 2:**
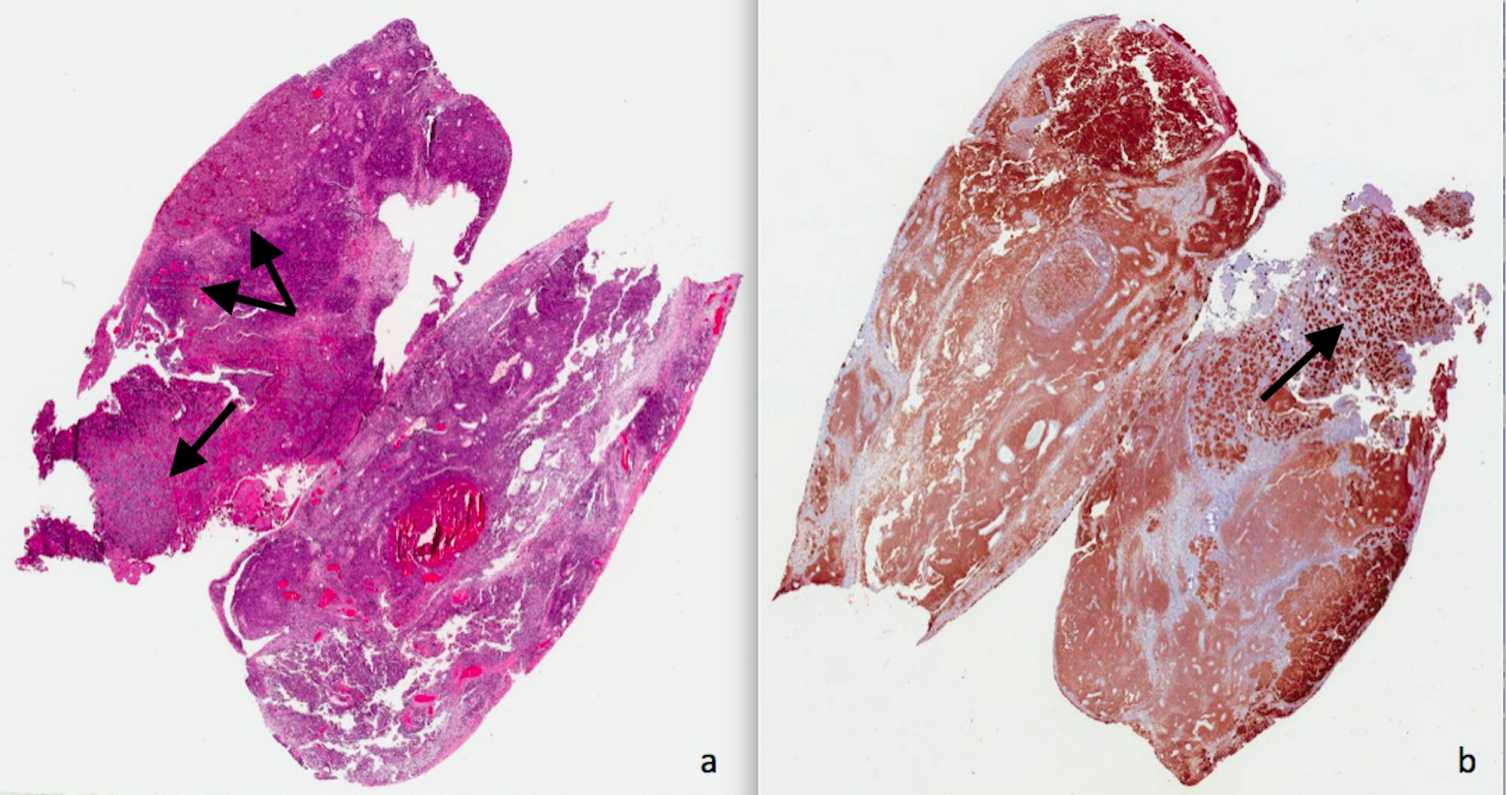
Photomicrographs of intradural extramedullary metastatic melanoma (H & E 25x). (a) Hypercellular tissue of spindle-shaped pattern of cells is seen with an epithelioid pattern with abundant vascularity. (b) The tissue is diffusely positive with immunostaining of a pan-melanoma cocktail (HMB45, MART-1, and tyrosinase). Immunoperoxidase staining for EMA, GFAP, CK7, and CK20 were all negative, consistent with melanoma. The tumor cells stained negative for cytokeratin AE1/AE3 and epithelial membrane antigen and stained positive (not shown) for S-100 and HMB45.

The patient underwent resection of the C1/C2 and C7/T1 spinal masses during the same procedure. The lesions were rubbery, tan, and hemorrhagic; and they were readily separable from the neural elements and dura. Pathology confirmed a diagnosis of melanoma (Figure 2). A fusion was also performed at the C6-T1 laminectomy site to prevent postoperative deformity. Two weeks later, the patient underwent craniotomy for resection of the temporal lobe lesion. The patient tolerated the procedures well and had a mild improvement in motor and sensory functions but not sufficiently enough to become ambulatory. At six months follow-up, the patient had sustained improvement in neurological function, but had developed visceral metastatic disease.

## Discussion

Malignant melanoma may present with intracranial disease as intracerebral hemorrhage, granulomatous meningitis, or localized symptoms secondary to the mass effect of the tumor. Spinal cord involvement is rare, however, with several cases of intramedullary spinal cord MM reported [8]. Chow et al. reported 10 IDEM tumors out of 266 spinal surgeries for spinal neoplastic disease, of which none were found to be MM [7]. Bullard et al. reported a large series of 1,341 patients with MM, eight of whom had spinal metastasis; however, it is not noted if any involved the IDEM space [9]. A review of the literature, however, demonstrates six definitive cases of MM metastatic to the intradural extramedullary space [7-8, 10-12]. This report is the first description of an initial presentation of MM presenting as a symptomatic spinal cord compression from tumor metastatic to the IDEM space.

The pathogenesis of intradural extramedullary metastatic disease is thought to mainly result from cerebrospinal fluid dissemination from cerebral lesions which are typically synchronous or antecedent to the spinal lesion, though hematogenous or lymphatic routes of entry are also postulated [7]. Gravity drainage of malignant cells in the subarachnoid space would explain the three separate tumor deposits in the spine in this case, and the particularly thick tumor deposition in the distal, lumbar sac. Leptomeningeal spread and extramedullary deposit of melanoma are typically late manifestations of disease and denote a grave prognosis.            

Although rare, MM to the spinal intradural extramedullary compartment may cause spinal cord compression, and this report documents that myelopathy may be the initial manifestation of disease. The findings in this case of thick matting of the cauda equina in the lumbosacral region by the tumor, multiple spinal foci of intradural deposits of tumor, and an intra-parenchymal brain lesion support a mechanism of disease dissemination through the cerebrospinal fluid. This report provides an additional facet to the need to consider MM in the differential diagnosis of an intradural lesion, particularly in patients with risk factors for or a history of melanoma.

## Conclusions

Metastatic melanoma should be considered in the differential of myelopathy secondary to a spinal intradural mass, particularly in those with a history of or risk factors for melanoma. An extensive work-up for malignancy is indicated in any patient presenting with findings of an intradural extramedullary mass lesion. Although generally a presentation of late disease, MM can present with CNS involvement as an initial presentation of disease.
